# IL-2 production correlates with effector cell differentiation in HIV-specific CD8+ T cells

**DOI:** 10.1186/1742-6405-3-18

**Published:** 2006-07-21

**Authors:** Laurel E Nomura, Brinda Emu, Rebecca Hoh, Perry Haaland, Steven G Deeks, Jeffrey N Martin, Joseph M McCune, Douglas F Nixon, Holden T Maecker

**Affiliations:** 1 BD Biosciences, Immunocytometry Systems, 2350 Qume Dr., San Jose, CA 95131, USA; 2 Division of Experimental Medicine, University of California, San Francisco, CA 94110, USA; 3 Department of Medicine, San Francisco General Hospital, University of California, San Francisco, CA 94110, USA; 4 BD Technologies, 21 Davis Dr., Research Triangle Park, NC 27709, USA; 5 Department of Epidemiology and Biostatistics, University of California, San Francisco, CA 94143, USA

## Abstract

**Background:**

Diminished IL-2 production and lack of effector differentiation have been reported for HIV-specific T cells. In this study, we examined the prevalence of these phenomena using 8-color cytokine flow cytometry, and tested the hypothesis that these two findings were causally related. We analyzed cytokine profiles and memory/effector phenotypes of HIV-specific and CMV-specific T cells using short-term *in vitro *stimulation with HIV or CMV peptide pools. Nineteen HIV-positive subjects with progressive disease and twenty healthy, HIV-negative subjects were examined.

**Results:**

Among HIV-infected subjects, there were significantly fewer CD8+ IL-2+ T cells responding to HIV compared to CMV, with no significant difference in CD4+ IL-2+ T cells. The majority of CMV-specific T cells in both HIV-negative and HIV-positive subjects appeared to be terminally differentiated effector cells (CD8+ CD27- CD28- CD45RA+ or CD8+ CD27- CD28- CD45RA-). In HIV-positive subjects, the most common phenotype of HIV-specific T cells was intermediate in differentiation (CD8+ CD27+ CD28- CD45RA-). These differences were statistically significant, both as absolute cell frequencies and as percentages. There was a significant correlation between the absolute number of HIV-specific CD8+ IL-2+ T cells and HIV-specific CD8+ CD27- CD28- CD45RA+ terminal effector cells.

**Conclusion:**

IL-2 production from antigen-specific CD8+ T cells correlates with effector cell differentiation of those cells.

## Background

The phenotype of CD4+ and CD8+ T cells responding to pathogens such as HIV or CMV is at least partially linked with their functions, which include cytokine production and cytotoxicity. In chronic HIV infection, the functional profile of HIV-specific T cells has been reported to be impaired in a variety of ways, including the ability to produce IL-2 [1-10]. This defect has been reported to apply to CD4+ [1,2,4,5,9,11] and CD8+ [3,6-8,10] T cells by different investigators.

Other studies have reported differences in the phenotype of HIV-specific CD8+ T cells compared to CMV-specific CD8+ T cells in subjects with chronic HIV infection [12-18]. In particular, a disproportionate number of cells of "intermediate" differentiation can be found among HIV-specific CD8+ T cells [12,17,18]. These "intermediate" cells have been variously described as CD27+ CD28- [12] and CCR7- CD45RA- [17,18], whereas most CMV-specific CD8+ T cells are terminally differentiated effector cells (CCR7- CD27- CD28- CD45RA+). A similar phenomenon of incomplete differentiation has been described for HIV-specific CD4+ T cells [19]. However, the prevalence of these differentiation "defects" among HIV+ individuals with progressive disease has not been well-defined. Furthermore, the relationship between differentiation state and IL-2 production has not been explored for either CD4+ or CD8+ HIV-specific T cells.

IL-2 is important for the survival and proliferation of activated T cells (reviewed in [20]). However, it has also been hypothesized to contribute to the differentiation of terminal effector CD8+ T cells in acute hepatitis C infection [21]. We reasoned that it was possible that a similar relationship might exist in chronic HIV infection, such that the independently observed defects in IL-2 production and differentiation of HIV-specific T cells might be associated.

To test this hypothesis, we simultaneously analyzed the cytokine production and phenotype of HIV-specific and CMV-specific T cells from a cohort of HIV-positive subjects with progressive disease, as well as CMV-specific T cells from HIV-negative subjects. We did short-term stimulation of PBMC with mixtures of peptides spanning multiple immunogenic proteins from HIV or CMV, then did a combined analysis for IFNγ and IL-2, as well as for CD27, CD28, and CD45RA. Results for each possible phenotype of cytokine-positive cells were expressed both as a percentage of CD4+ or CD8+ T cells and as an absolute number of cells per ml. Our simultaneous analysis of differentiation and function of HIV-specific and CMV-specific T cells allowed for the ability to see whether changes in differentiation were correlated with changes in function, for either CD4+ and/or CD8+ T cells.

## Results

### Stability of effector/memory markers in short-term stimulation

**Figure 1:**
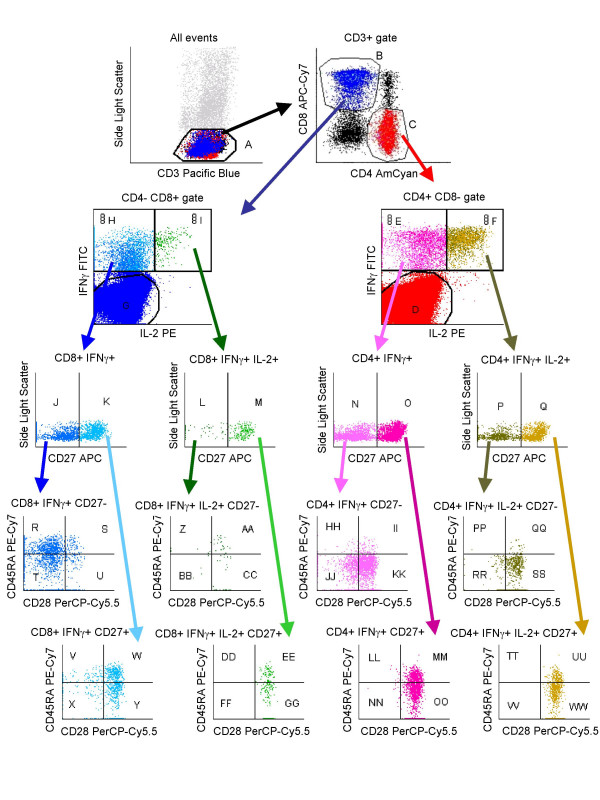
Hierarchical gating strategy. After applying an acquisition threshold on CD3, gate A was constructed to include CD3 dim cells. CD3+ low-scatter cells were identified either as CD4+ CD8- or CD4- CD8+ (gates B and C, which also included dim positive cells). Cytokine+ cells in gates B and C were identified either as total IFNγ+ or IFNγ+ IL-2+. These two classes of cytokine-producing cells were determined to be either CD27- or CD27+, and then further separated by expression of CD28 and CD45RA. Gates R through WW represent the 32 ultimate phenotypes compared in this study. The example shown is from a pp65+IE-1 stimulated HIV-negative subject.

**Figure 2:**
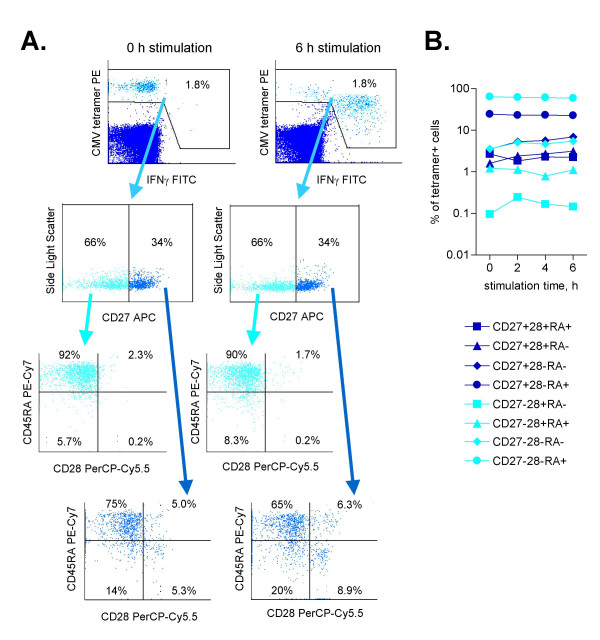
Effects of stimulation on memory cell markers. A CMV-positive whole blood sample from a healthy donor was incubated for six hours with pp65 peptide, in the presence of brefeldin A. Phenotypic analysis was performed both before and at various time points during stimulation. A: Gating on CMV tetramer-positive cells, the response to stimulation after six hours is demonstrated by the release of cytokine. By isolating the tetramer-positive IFNγ+ cells, it is evident that there are only nominal changes in the proportions of memory cell markers after six hour stimulation. B: Changes in each individual phenotype over time are shown. Results are expressed as absolute number of cells per milliliter of blood.

We simultaneously analyzed cytokine production and effector/memory markers of CMV- and HIV-responsive T cells using the gating strategy shown in Figure 1, in a cohort of 19 HIV-positive subjects with progressive disease and 20 healthy controls (Table 1). Since activation of T cells was required to detect cytokine production, we wanted to ensure that the phenotypic markers examined did not modulate during short-term stimulation. Using an MHC-peptide tetramer to isolate CMV-specific T cells, we demonstrated that 6-hour stimulation had minimal effect on the distribution of CD27, CD28, and CD45RA on these cells (Figure 2). Therefore, our analysis closely reflected the *in vivo *state of differentiation of these cells, rather than the effects of *in vitro *stimulation.

**Table 1:** Subject characteristics

HIV-negative subjects			HIV-positive subjects				
Subject	Abs CD4^1^	Abs CD8^1^	Subject	Abs CD4^1^	Abs CD8^1^	Viral Load^2^	ART^3^

101	1382	1233	1026	299	304	69200	No
105	787	687	1027	543	978	36600	No
107	490	323	1034	330	609	29400	No
111	541	236	1036	433	1259	4880	No
114	686	327	1038	17	159	>500000	No
119	497	184	1059	417	1628	21500	No
120	697	364	1079	381	756	76200	No
121	882	341	1501	493	648	18100	No
124	828	1101	1503	528	1094	8000	No
133	943	900	2008	333	499	5600	No
137	591	516	3002	158	456	21700	No
138	1278	612	3012	312	1138	9000	Yes
142	820	577	3067	60	928	27400	No
144	523	637	3093	560	2431	7300	Yes
148	445	416	3094	190	837	12800	Yes
150	795	868	3102	104	1548	37200	Yes
156	983	951	3114	43	575	16100	No
157	828	766	3114	26	385	9400	No
158	598	525	3168	110	1676	262000	Yes
1075	1092	270	3170	192	750	28200	Yes
					
mean	784^4^	592^5^	mean	276^4^	933^5^		

^1^Abs = Absolute Cell Count, as number of cells per microliter of blood.

^2^Viral Load, as copies per milliliter of blood.

^3^ART = Currently receiving anti-retroviral therapy.

^4^Absolute CD4 cell counts of HIV- and HIV+ subjects are significantly different (*p *< 0.0001).

^5^Absolute CD8 cell counts of HIV- and HIV+ subjects are significantly different (*p *= 0.0439).

### Decreased IL-2 production in HIV-specific CD8+ but not CD4+ T cells

**Figure 3:**
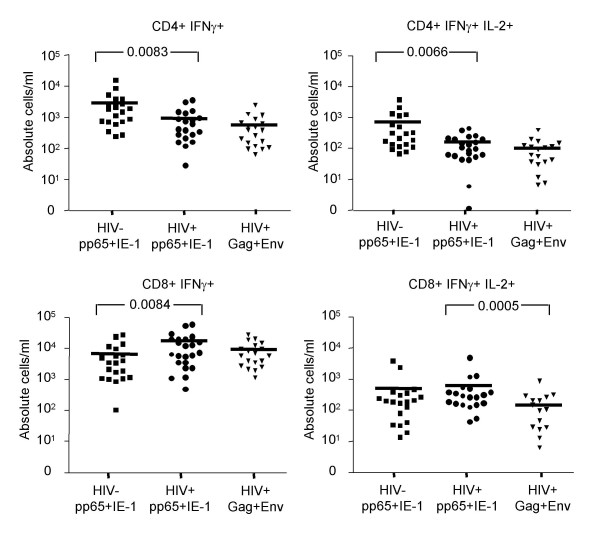
Responses of cytokine-specific T cells to CMV and HIV stimulation. Each symbol represents one patient; mean response is shown as a horizontal line. Results are displayed as absolute number of relevant cells (CD4+ IFNγ+, CD4+ IFNγ+ IL-2+, CD8+ IFNγ+, or CD8+ IFNγ+ IL-2+) per ml of blood. Subjects and stimuli are listed below the X axis. Statistical comparisons between HIV-positive and HIV-negative subjects were performed using a Mann Whitney test. Statistical comparisons between different stimulations of HIV-positive samples were performed using a Wilcoxon signed rank test. Values of *p *< 0.025 were considered statistically significant.

The magnitude of the T cell IFNγ responses to CMV pp65+IE-1 and to HIV Gag+Env, as well as the IL-2+ component of those responses, is shown in Figure 3 for 20 healthy HIV-negative subjects and 19 HIV-positive progressors. There were significant differences in the magnitude of CMV responses of HIV-negative versus HIV-positive subjects. These were attributable to differences in CD4 and CD8 counts between the groups (see next paragraph). However, the only significant difference in the magnitude of CMV versus HIV responses of HIV-positive subjects was a reduced frequency of HIV-specific CD8+ T cells producing both IFNγ and IL-2 (*p *= 0.0005).

**Figure 4:**
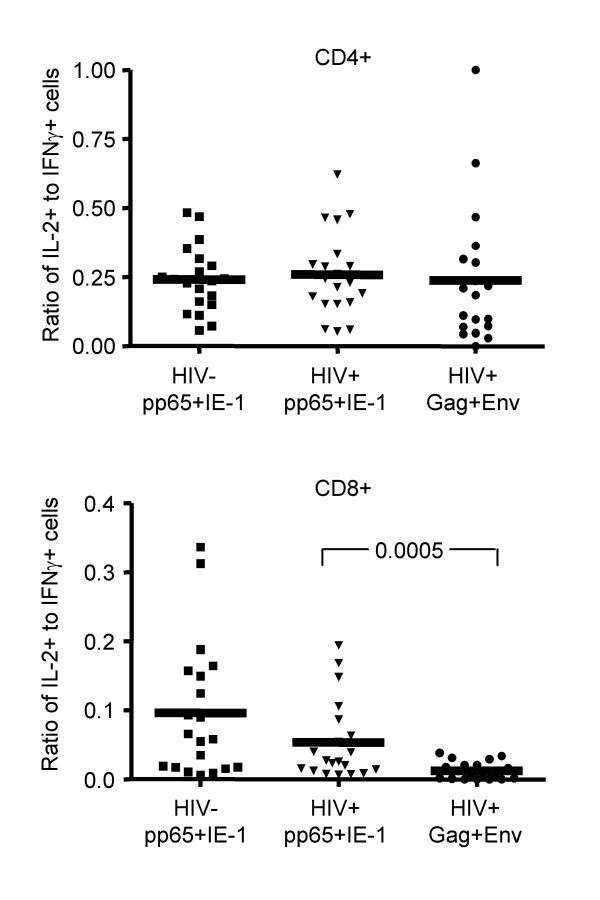
Ratio of IL-2+ to IFNγ+ T cells responding to HIV versus CMV. Each symbol represents one patient; the mean response is shown as a horizontal line. Subjects and stimuli are listed below the X axis. Statistical comparisons between HIV-positive and HIV-negative subjects were performed using a Mann Whitney test. Statistical comparisons between different stimulations of HIV-positive samples were performed using a Wilcoxon signed rank test. Values of *p *< 0.025 were considered statistically significant.

To determine whether this reduction in CD8+ T cell IL-2 production among HIV-specific T cells was consistent when data were analyzed on a proportional basis, we examined the ratio of IL-2+/IFNγ+ T cells responding to HIV versus CMV (Figure 4). This analysis clearly showed that the proportion of HIV-specific CD8+ T cells that could produce IL-2 was significantly reduced compared to that of CMV-specific CD8+ T cells in HIV-positive subjects (*p *= 0.0005). No other significant differences in IL-2+/IFNγ+ T cell ratios were found.

### Incomplete differentiation in HIV-specific CD4+ and CD8+ T cells

We next analyzed the differentiation profile of HIV-specific and CMV-specific T cells in healthy subjects versus HIV-positive progressors. Several trends were observed.

**Figure 5:**
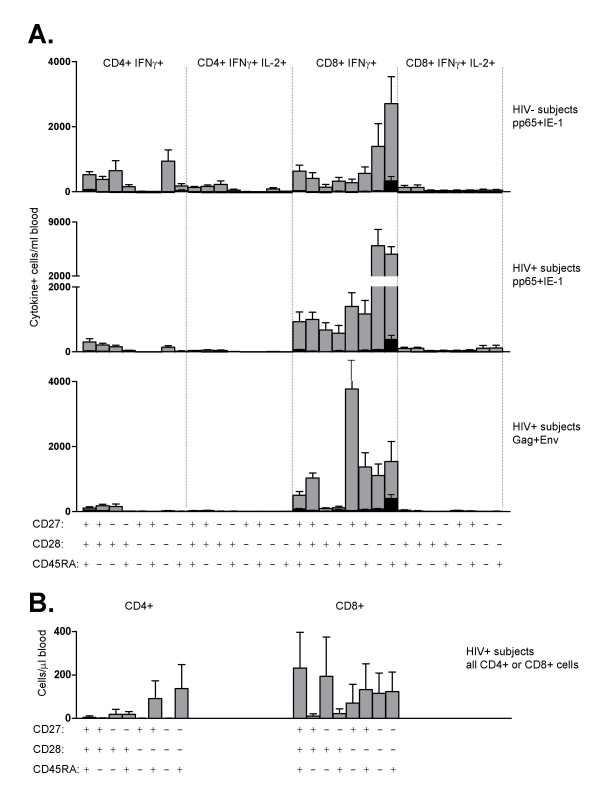
A: Individual phenotypic responses of HIV-negative and HIV-positive subjects to pp65+IE-1 or Gag+Env stimulation. Grey bars represent the mean (for 20 HIV-negative or 19 HIV-positive subjects) of the absolute number of cells for each of the 32 phenotypes, as listed at the bottom of the figure. Black bars represent the background, as mean of the absolute number of cytokine-positive cells in the unstimulated samples, for each phenotype. In both cases, error bars represent SEM. Phenotypes are grouped horizontally as CD4+ or CD8+, and by their cytokine profile. Within each subgroup, phenotypes are arranged from naïve (left) to terminal effector (right), with intermediates arranged by order of potential for IL-2 production in healthy donors (see Figure 6, top panel). Results are shown for the response of HIV-negative subjects to pp65+IE-1 stimulation (top), the response of HIV-positive subjects to pp65+IE-1 stimulation (middle), and the response of HIV-positive subjects to Gag+Env stimulation (bottom). B: Individual phenotypes of total CD4+ or CD8+ T cells. Each bar represents the mean (for 19 HIV-positive subjects) of the absolute number of cells displaying each of the phenotypes, as listed at the bottom of the figure. Error bars represent SEM. Results are shown for HIV-positive patient PBMC stimulated with Gag+Env peptide mix, for direct comparison to the HIV-specific response shown in A.

The response of healthy subjects to CMV (Figure 5A, top panel) was quite heterogeneous, involving most of the possible phenotypes of both CD4+ and CD8+ T cells. However, the CD8+ response was higher than the CD4+ response, and the fraction of IFNγ+ IL-2+ T cells was small compared to total IFNγ+ T cells. Among the CD8+ T cell phenotypes, terminally-differentiated effector cells predominated, followed by CD27- CD28- CD45RA- cells. Thus, the healthy donor response to CMV was highly skewed towards effector cell phenotypes.

The CMV-specific response of HIV-positive progressors was similarly skewed towards effector CD8+ IFNγ+ T cells (Figure 5A, middle panel). The number of CMV-specific CD4+ T cells was significantly lower in the HIV-positive subjects, although the distribution of phenotypes was similar to that of HIV-negative subjects. These differences were largely a result of reduced CD4+ T cell counts in the HIV-positive subjects, since the differences were not statistically significant on a percentage basis (Table 2).

**Table 2:** Analyses of variance

		Absolute Count		Percent CD4+ or CD8+ T cells	
		
Subjects	Population	pp65+IE-1 stimulation, HIV- vs HIV+ subjects	pp65+IE-1 vs Gag+Env stimulation, HIV+ subjects	pp65+IE-1 stimulation, HIV- vs HIV+ subjects	pp65+IE-1 vs Gag+Env stimulation, HIV+ subjects
All	(CD4+ IFNγ+)	**<.00001**^1^	**0.00120**	0.08193	0.11850
	(CD4+ IFNγ+ IL-2+)	**<.00001**	**0.00310**	0.99855	**0.01903**
	(CD8+ IFNγ+)	**0.00001**	**0.00002**	0.05149	**<.00001**
	(CD8+ IFNγ+ IL-2+)	0.82219	0.06415	0.80507	**0.00760**
					
Non-ART only	(CD4+ IFNγ+)	**<.00001**	**0.00016**	0.05508	**0.01023**
	(CD4+ IFNγ+ IL-2+)	**<.00001**	**0.00184**	0.98033	**0.02026**
	(CD8+ IFNγ+)	0.99841	**0.00001**	0.94575	**0.00008**
	(CD8+ IFNγ+ IL-2+)	0.18590	0.09745	0.57196	0.05185

^1 ^All results are expressed as p-values. p < 0.05 was considered significant (shown in bold).

The HIV response of HIV-positive progressors (Figure 5A, bottom panel) was different from the CMV responses in that it was dominated by CD8+ IFNγ+ T cells of intermediate differentiation (CD27+ CD28- CD45RA-). This phenotype was rare within the healthy donor CMV response. Also, the HIV responses contained hardly any CD4+ or CD8+ IL-2-producing cells, and only very few CD4+ IFNγ+ cells. These differences in CD4 and CD8 compartments were significant by ANOVA (Table 2), with the most highly significant differences being in the CD8+ IFNγ+ subset (*p *≤ 0.00008). These differences were significant even when subjects receiving ART were excluded (Table 2, bottom half).

It should be noted that the distribution of CD8+ T cell phenotypes seen in HIV-responsive cells was not reflected in the overall CD8+ T cell compartment (Figure 5B). The total CD8+ T cell pool was quite heterogeneous, and included a large cohort of effector-like cells (CD27- CD28- CD45RA+ and CD27- CD28- CD45RA-).

Overall, the data of Table 2 confirm that the differences in phenotypic patterns observed in the CMV and HIV responses of HIV-positive progressors were statistically significant. The most significant difference (*p *≤ 0.00008) was seen in CD8+ IFNγ+ cells. This was true when data were analyzed as percentages or as absolute counts, and whether or not subjects on ART were included.

### Relationship of phenotype to IL-2 production

The above data demonstrate that HIV-specific CD8+ T cells in HIV-positive progressors show two major differences compared to CMV-specific CD8+ T cells: (1) a lower proportion of IL-2-producing cells, and (2) a less differentiated phenotype. We tested two potential hypotheses that might explain the coexistence of these two phenomena.

**Figure 6:**
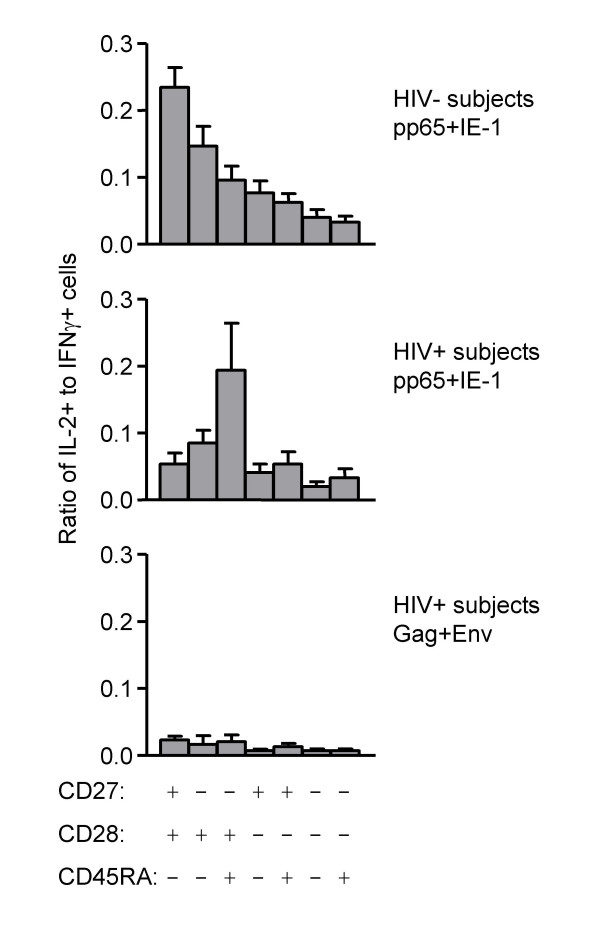
Production of IL-2 by various phenotypes of CD8+ T cells. Each bar represents the mean (for 20 HIV-negative or 19 HIV-positive subjects) of the ratios of IL-2 to IFNγ production for each of the phenotypes, as listed at the bottom of the figure. Phenotypes are arranged by amount of IL-2 production; naïve cells (CD27+ CD28+ CD45RA+) were not analyzed as they are not expected to make IFNγ. Error bars represent SEM. Results are shown for the response of HIV-negative subjects to pp65+IE-1 stimulation (top), the response of HIV-positive subjects to pp65+IE-1 stimulation (middle), and the response of HIV-positive subjects to Gag+Env stimulation (bottom).

The first hypothesis, that the less differentiated CD8+ T cells tend not to produce IL-2, can be easily dismissed by examination of IL-2 production as a function of phenotype (Figure 6). While IL-2-producing T cells could be found amongst all phenotypes of CD8+ IFNγ+ T cells specific for CMV, proportionally fewer cells produced IL-2 upon differentiation to effector phenotypes (Figure 6, top panel). This pattern was somewhat disrupted in the CMV response of HIV-positive subjects (middle panel), but the most pronounced difference was observed in the HIV response (bottom panel). HIV-responsive CD8+ T cells of all phenotypes essentially did not make IL-2. Thus, there is a pervasive breakdown in IL-2 production from all HIV-specific CD8+ T cells, and not simply a replacement of cells that normally make IL-2 with cells that normally do not.

### HIV-specific defects in differentiation and function are correlated

**Figure 7:**
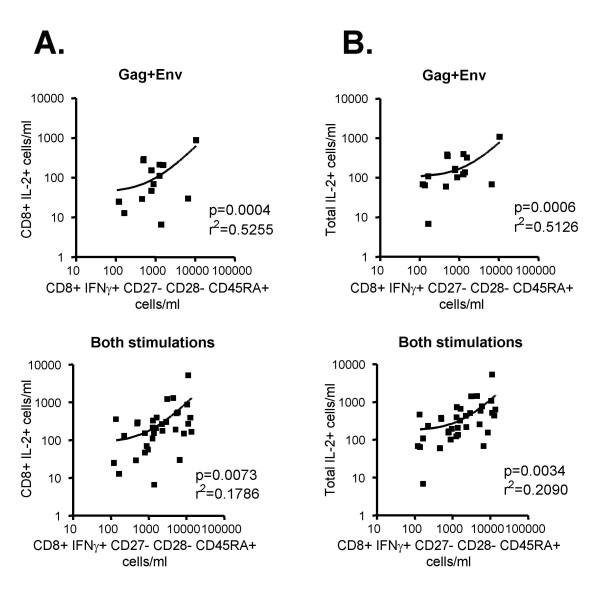
Correlation analyses of IL-2 production and effector cell differentiation. A. Top, the absolute number of the HIV-specific CD8+ IFNγ+ IL-2+ T cells is plotted against the absolute number of the HIV-specific CD8+ terminal effector T cells (CD27- CD28- CD45RA+); bottom, the same correlation, but including CMV-specific responses of the same subjects. B. Top, the absolute number of all HIV-specific IFNγ+ IL-2+ cells (both CD8+ and CD4+) is plotted against all HIV-specific terminal effector T cells; bottom, the same correlation, but including CMV-specific responses of the same subjects. In all correlations, each square represents a single subject's CMV or HIV response.

A second hypothesis for the co-existence of fewer CD8+ IL-2+ T cells and altered differentiation in HIV responses was that IL-2 production is required to drive effector T cell differentiation. If this were true, one would expect to see a quantitative correlation between CMV-specific or HIV-specific T cells and differentiation state. Figure 7A shows that the number of HIV-specific IFNγ+ IL-2+ CD8+ T cells is significantly correlated with the number of HIV-specific CD8+ terminal effector T cells (CD27- CD28- CD45RA+) (*p *= 0.0004, top). The correlation remained significant when CMV-specific responses were also included (*p *= 0.0073, bottom). Finally, the correlations remained significant (*p *= 0.03) when frequencies were reported as a percentage of CD8+ T cells, rather than as absolute counts (data not shown). Therefore, a greater number of CD8+ IL-2-producing cells correlated with more terminally differentiated CD8+ effector cells in the antigen-specific response.

We also looked at the correlation of CD4+ IL-2-producing cells with CD8+ effector cells. These were not as well correlated for either HIV-specific or pooled HIV-specific and CMV-specific responses (*p *= 0.0743 and *p *= 0.0410, respectively; data not shown). However, when the frequency of all IFNγ+ IL-2+ T cells (CD4+ and CD8+) were plotted against the frequency of CD8+ terminal effector cells (Figure 7B), the correlation was highly significant, both for the HIV-specific stimulation (*p *= 0.0006, top) and for pooled HIV-specific and CMV-specific results (*p *= 0.0034, bottom). This argues strongly for a role of IL-2 in promoting CD8+ effector T cell differentiation.

## Discussion

In this study, we conducted a detailed analysis of the phenotypes and functions of HIV-specific and CMV-specific T cells in subjects with progressive HIV disease, compared to healthy subjects. By using pools of peptides representing multiple HIV and CMV antigens, we were able to analyze a large proportion of the total virus-specific response, rather than analyzing only single epitope responses. By pooling the results from approximately 20 subjects in each group, we reduced bias due to individual differences within groups, which were large. By determining T-cell phenotypes using a comprehensive gating hierarchy, we were able to classify every cell according to one of 32 unique differentiation profiles. This allowed a more standardized and complete approach than could be achieved by comparing only a few markers at a time.

By expressing results as absolute counts of CD4+ or CD8+ T-cells, we accounted for the wide variety of absolute CD4+ T cell counts among our subjects (Table 1). Nevertheless, significant differences between HIV and CMV responses of HIV-positive progressors remained when data were analyzed as percentages of CD4+ or CD8+ T cells (Table 2).

The results of our analysis confirm and extend findings that were previously reported in separate investigations: namely, that HIV-specific CD8+ T cells producing IL-2 are reduced in number and proportion compared to CMV-specific CD8+ T cells producing IL-2 [3,6-8], and that effector cell differentiation of CD8+ T cells differs between HIV and CMV responses [12-17].

Our observation of reduced CD8+ T cell IL-2 production is consistent with published reports about the lack of proliferative capacity of these cells in progressive HIV disease [6,15,22]. However, we did not see a similar loss in IL-2-producing CD4+ T cells specific for HIV as compared to CMV, which has also been previously reported [1,2,4,5,9]. This could be due to our reporting of these cells on an absolute count basis. But we also observed no significant differences in the ratio of IL-2+/IFNγ+ CD4+ T cells between CMV and HIV responses of HIV-positive progressors. In general, the CD4 responses of this cohort were very low.

The CMV response of healthy subjects showed the highest degree of effector cell differentiation among CD8+ T cells, and these subjects tend to have undetectable CMV viral loads in blood [23]. By contrast, high HIV viral loads were present in all of the HIV-positive subjects (Table 1). Yet, these subjects tended to have fewer CD8+ effector T cells and more cells of intermediate differentiation. Thus, the differentiation of the CD4+ and CD8+ T cell response to HIV can be thought of as defective in HIV-positive progressors, in that it does not reflect viral load, and does not result in the effector cells that are associated with control of CMV viral load in healthy subjects.

We did not observe a significant relationship between HIV viral load and CD4+ or CD8+ T cell differentiation status (data not shown). This is in contrast to studies that showed a relationship between CD4+ T cell differentiation and viral load [2,4,24]. However, these studies all involved long-term non-progressors or others who controlled viremia, whereas the cohort in the present study consisted entirely of progressors with viral loads >4800. Thus, it is certainly plausible that T cell differentiation becomes "uncoupled" with viral load at some stage of HIV progression or level of persistent viremia. In fact, our results would argue that this may occur due to a defect in IL-2 production, which itself may be a result of persistently high antigen load [25,26].

A recent report has suggested that cells of earlier stages in CD8+ differentiation (CD28+) are important for control of CMV [27]. By analogy, HIV-specific CD8+ T cells can also be thought of as defective, since they are predominantly CD27+ CD28- CD45RA-. However, the proportion of all CD8+ CD28+ T cells responding to HIV was not significantly different from that responding to CMV in our study (data not shown). Our data thus do not support a universal role for CD8+ CD28+ T cells in control of chronic viral infection.

One might hypothesize that the altered differentiation of HIV-specific T cells we observed might be related to disease progression as measured by absolute CD4 count. However, there was no significant correlation between CD4 count and CD4+ or CD8+ T cell differentiation status in this study (data not shown). This suggests that altered differentiation of HIV-specific T cells may be an additional marker of disease progression, independent of CD4 count and viral load. In fact, the relationship of viral load, CD4 count, and HIV-specific T cell responses is complex [25,26], and large prospective studies will be necessary to determine causal relationships between them.

Because our analysis combined phenotypes and function, we were able to ask whether phenotypic and functional defects were quantitatively related. While a clear relationship between phenotype and IL-2 production could be seen in CMV-responsive CD8+ T cells of healthy donors (Figure 6), that relationship was disrupted in HIV responses. These latter displayed a pervasive lack of IL-2 production among CD8+ T cells of all phenotypes. Nevertheless, there was a significant correlation between the number of CD8+ IL-2+ T cells and the number of CD8+ terminal effector T cells (CD27- CD28- CD45RA+) responding to HIV (Figure 7). This is despite the fact that the terminal effector cells are least likely to actually produce IL-2 (Figure 6). In fact, IL-2 production was most correlated with CD28 expression, among the three memory/effector markers used in this study (data not shown), in agreement with previous work demonstrating that CD28 expression is important for IL-2 production [3]. The correlation of terminal effector cell numbers with IL-2-producing cells is thus unexpected.

## Conclusion

In this paper, we have confirmed two important defects in cellular immunity to HIV that were previously reported only in separate studies. Furthermore, we have unexpectedly shown that these two defects are quantitatively correlated, suggesting a mechanistic involvement of IL-2 production in the differentiation of CD8+ effector T cells. This has implications for immunotherapy and immunomonitoring of HIV disease, as it suggests that the preservation of HIV-specific CD8+ IL-2 production is likely to be important for maintaining effector T cell-mediated viral control.

## Methods

### Study subjects

#### HIV-positive subjects

Nineteen subjects (called "progressors" in this study) were selected from an ongoing study at the San Francisco General Hospital and San Francisco Veterans Affairs Medical Center (Study of the Consequences of the Protease Inhibitor Era [SCOPE]). Progressors (Table 1) had a decrease in CD4+ T cell counts to less than 500 cells/mm^3 ^during the chronic stage of their infection, and had persistent plasma HIV RNA levels >2000 copies/ml. Current CD4 count, duration of therapy, specific therapies, and self-reported duration of HIV infection were not factors for exclusion from this study. All HIV-positive subjects were also CMV-positive.

#### HIV-negative subjects

Twenty CMV-positive subjects (Table 1) were selected from BD Biosciences' in-house pool of blood donors. All subjects were adults and were asymptomatic and healthy at the time of sample collection.

Informed consent was obtained from subjects, and human experimentation guidelines of the US Department of Health and Human Services and of all involved institutions were followed.

### Viral load and absolute cell count determination

#### HIV-positive subjects

Plasma HIV RNA levels were determined by the branched DNA (bDNA) amplification technique (Quantiplex^® ^HIV RNA, version 3.0, Chiron Corporation, Emeryville, CA). CD4 and CD8 counts were obtained using the Multitest assay with a FACSCalibur flow cytometer and Multiset software (BD Biosciences, San Jose, CA).

#### HIV-negative subjects

CD4 and CD8 absolute cell counts were determined using TruCount "Hi" beads and TriTest CD3 FITC/CD4 PE/CD45 PerCP antibodies (BD Biosciences) [28].

### Sample preparation and activation

Individual peptides of 15 amino acid residues, overlapping by 11 amino acids each, were designed to span the sequences of CMV pp65 (138 peptides) and IE-1 (Immediate Early-1; 120 peptides), and HIV p55 Gag (SF2 strain, 127 peptides) and env (MN strain, 204 peptides). Peptide mixes (SynPep, Dublin, CA) were dissolved in DMSO at stock concentrations of 0.7–1.0 mg/ml per peptide. The mixes were used at a final concentration of 1.2–2 μg/ml per peptide [29].

PBMC were isolated from heparinized whole blood within eight hours of collection, by centrifugation in CPT tubes (BD Vacutainer, Franklin Lakes, NJ). PBMC were washed and resuspended in RPMI with 10% fetal bovine serum (cRPMI) at 5 × 10^6 ^– 1 × 10^7 ^cells/ml. Five hundred microliters of PBMC in cRPMI were aliquoted into wells of a 24-well deep-well plate (Qiagen, Valencia, CA). CMV pp65+IE-1 or HIV p55 Gag+Env peptide mixes were added to the appropriate wells, plus brefeldin A (BD Biosciences) at a final concentration of 10 μg/ml; an additional well received only brefeldin A as a negative control. The plate was incubated for six hours at 37°C, then held overnight at 18°C.

### Sample processing and staining

Cell-surface markers such as CD3, CD4, and CD8 can be stained before or after cell fixation and permeabilization. We stained for these markers before fixation, since this enhanced resolution of positive and negative populations. Although some down-modulation of these markers occurred on activated cells, we were still able to include down-modulated cells within the positive gate.

Details of the cytokine flow cytometry protocol can be found on the Maecker Lab weblog [30]. Briefly, cells were stained for 60 minutes with CD28 PerCP-Cy5.5, CD45RA PE-Cy7, CD27 APC, CD8 APC-Cy7, CD3 Pacific Blue, and CD4 AmCyan (all from BD Biosciences). Samples were treated with BD FACS Lysing Solution followed by BD FACS Permeabilizing Solution 2, washed, then stained with IFNγ FITC and IL-2 PE (BD Biosciences) for 60 minutes. They were then washed and resuspended in BD Stabilizing Fixative (BD Biosciences) and held at 4°C until sample acquisition.

For each donor, 100 μl of PBMC remained unstained, for use when establishing PMT voltage settings. Eight tubes containing BD CompBeads Anti-Mouse Ig,κ and Negative Control Compensation Particles (BD Biosciences) were each stained with one of the eight mAbs listed above. The beads were treated identically to the PBMC samples, except that all stains (including cytokines) were added during the "surface staining" step.

### Sample acquisition and analysis

Flow cytometry was performed using an LSRII (BD Biosciences) equipped with blue (488 nm), red (633 nm), and violet (405 nm) lasers. Compensation settings were established using the AutoComp option of FACS DiVa software (BD Biosciences). An average of 2 × 10^6 ^CD3+ cells were collected for HIV-negative subject samples, and an average of 4 × 10^5 ^CD3+ cells were collected for HIV-positive subject samples.

Data were analyzed with FACS DiVa software. Where appropriate, data were displayed with a transformation that allows for improved visualization of events at the lower end of the log scale (BiExponential analysis). A gating hierarchy was designed as shown in Figure 1. The result of this gating strategy was 32 possible phenotypes of antigen-responsive T cells.

Dead cells were excluded by use of a side scatter gate and by gating on single-stained cells for either CD4 or CD8. Non-viable cells generally bound both of these antibodies non-specifically.

Cytokine production in the absence of stimulation was low, and largely restricted to CD8+ terminally differentiated effector cells (CD27- CD28- CD45RA+ [12,15,31-37]; Fig 5A, black bars). As such, backgrounds were not subtracted from the responses shown in the grey bars. Responses ≥ 100 cells/ml were significantly higher than background for all phenotypes except the terminal effector cells mentioned above.

Data were batch-analyzed to ensure uniform gating, and statistics files were uploaded to a database created for this study. This database enabled selection of particular data subsets of interest, which were then downloaded into Microsoft Excel and GraphPad Prism (GraphPad, San Diego, CA) for statistical analysis and graphing. Statistical comparisons between HIV-positive and HIV-negative subjects were performed using a Mann Whitney test. Statistical comparisons between different stimulations of HIV-positive samples were performed using a Wilcoxon signed rank test. Values of *p *< 0.025 were considered statistically significant, to correct for the two sets of comparisons listed above.

ANOVA (Table 2) were conducted using the Open Source statistics package R [38]. For each of the four cell populations (CD4+ IFNγ+, CD4+ IFNγ+ IL-2+, CD8+ IFNγ+, or CD8+ IFNγ+ IL-2+), a separate model was fit in which the response was the absolute cell count for each subject and the main effect was the eight combinations of the markers CD27, CD28, and CD45RA. Two contrasts were added to test specifically whether or not the average numbers for each phenotype followed the same pattern for HIV-positive CMV versus HIV-negative CMV, and for HIV-positive CMV versus HIV-positive HIV responses. The *p*-values reported were for the interaction term between each of these contrasts and the main effect for phenotype. A *p*-value < 0.05 indicates that the two subject groups had significantly different patterns in the numbers of cells of each phenotype.

## Declaration of competing interests

The authors declare that they have no competing interests, except that LN, PH, and HM are employees of Becton Dickinson, which manufactures reagents and equipment for HIV diagnostic testing.

## Authors' contributions

LN carried out all sample processing and analysis of data, and helped to draft the mauscript. HM conceived of the study, oversaw its design and coordination, and drafted the manuscript. PH carried out ANOVA and other statistical analyses. BE and RH coordinated collection of the HIV+ samples. SD, JNM, JMM, and DN created and managed the SCOPE study and helped to edit the manuscript. All authors read and approved the final manuscript.
